# Thiolated Chitosan-carboxymethyl Dextran Nanoparticles: Improving Intravitreal Drug Bioavailability for Retinoblastoma

**DOI:** 10.18502/jovr.v17i1.10163

**Published:** 2022-01-21

**Authors:** Lauren A Dalvin

**Affiliations:** ^1^Department of Ophthalmology, Mayo Clinic, Rochester, Minnesota, USA

In the current issue of *Journal of Ophthalmic and Vision Research* (*JOVR*), Delrish et al discuss the use of modified nanoparticles for intravitreal drug delivery in a rat model of retinoblastoma.^[1]^ Retinoblastoma is the most common intraocular malignancy of childhood, with an incidence of 11.8 cases per million children aged 0–4 years in the US and with recent increase in incidence reported in Europe.^[2,3]^ While developing countries often face more advanced disease and worse survival prognosis, retinoblastoma is considered curable, and mortality is rare in well-developed nations.^[4]^ Although the primary goal of retinoblastoma treatment is to protect the child's life, secondary and tertiary goals of globe salvage and visual acuity preservation have become more achievable with treatment advances, particularly the combination of intra-arterial and intravitreal chemotherapy. With current standard of care therapy, globe salvage can be achieved even for the most advanced group E eyes in about 50% of cases.^[5]^

Delrish et al describe what may be the next frontier in intravitreal chemotherapy delivery, with improved drug bioavailability using thiolated chitosan-carboxymethyl dextran nanoparticles (CMD-TCs-NPs). Although intravitreal injection offers direct drug access to the vitreous cavity and retina, it can take several hours for drug to diffuse across the entire vitreous cavity.^[6,7]^ Moreover, *in vitro *experiments show that drug dispersion in the vitreous cavity is driven by advective mass transport rather than molecular diffusion, relying on natural saccadic movements to distribute the drug.^[8,9]^ Drug distribution may be especially challenging in more formed pediatric vitreous, and speed of dispersion could be important to achieve tumor control in the setting of drugs such as melphalan, which have a short half-life.^[10]^ Thus, improved bioavailability of intravitreal chemotherapy agents could decrease injection burden and retinal toxicity, improve overall tumor control rates, and improve globe salvage for retinoblastoma with vitreous seeding.

Chitosan is a semi-synthetic cationic polysaccharide produced by partial or complete deacetylation of the naturally occurring polymer chitin, which can be harvested from crustacean shells or fungal cell walls.^[11,12]^ Chitosan is highly biocompatible, nontoxic, and mucoadhesive; has favorable enzymatic biodegradability, low immunogenicity, and inherent antibacterial properties; and can disrupt epithelial tight junctions to enhance permeability.^[13]^ Given these favorable properties, combined with easy accessibility, chitosan has become a popular candidate for pharmaceutical development, and the US Food and Drug Administration has already approved several chitosan-containing products.^[13]^

Mucoadhesive polymers like chitosan contain numerous charged molecular groups that can form non-covalent bonds with mucin, allowing bioadhesion to mucosal surfaces.^[13]^ Numerous factors can influence mucoadhesion, including pH, concentration, ionic strength, temperature, incubation time, surface charges, molecular weight, degree of deacetylation, and hydrophilicity, among others.^[13]^ Chitosan, in particular, has pH-dependent mucoadhesion, and certain modifications have been employed to improve the polymer's mucoadhesive properties at physiologic pH relevant to ophthalmic drug delivery. Trimethyl chitosan has been extensively studied and has strong mucoadhesive properties due to its high cationic charge. Thiolated chitosan derivatives have also been studied, which have free thiol groups to form covalent bonds with cysteine-rich subdomains of mucus glycoproteins.^[13]^ Compared to unmodified chitosan, thiolated chitosan has several advantages, including 6- to 100-fold improved mucoadhesion, 1.6- to 3-fold enhanced permeability, and *in situ* gelling properties at physiologic pH.^[14,15]^ Together, these properties improve drug residence time and increase bioavailability. Thiolated derivatives require sulfhydryl groups to react, limiting use to cysteine-rich tissues,^[13]^ but secreted protein acidic and rich in cysteine (SPARC) has demonstrated localization to Muller and ganglion cells in the retina, making the retina a reasonable target tissue.^[16]^

In ophthalmology, initial interests aligned with the use of chitosan to improve bioavailability of topically administered medications, but there is a growing body of literature on possible intravitreal applications.^[11]^ Previous studies have demonstrated that nanoparticle surface properties are a key factor determining distribution in the retina and vitreous following intravitreal injection, and although glycosylated chitosan polymers maintained a positive charge and could reach the retina, these particles were not able to penetrate the internal limiting membrane.^[17]^ Thiolated derivatives, on the other hand, may have improved penetration due to their interaction with cysteine-rich SPARC localized to Muller cells. In this issue of *JOVR*, Delrish et al specifically compare the biodistribution of thiolated versus methylated chitosan-carboxymethyl dextran nanoparticles (CMD-TCs-NPs vs CMD-TMC-NPs) following an intravitreal injection in a rat model of retinoblastoma.^[1]^ The authors found improved uptake of CMD-TCs-NPs in retinoblastoma cell culture, and in the rat model, 24 hr after injection; the CMD-TCs-NPs demonstrated diffusion throughout the vitreous cavity and retinal penetration, while the CMD-TMC-NPs were immobilized in the vitreous.^[1]^ The authors attributed these differences to improved bioadhesion of thiolated chitosan and prohibitive effects on vitreous diffusion of the greater positive charge of methylated chitosan (zeta potential +29mV) compared with the thiolated counterparts (+11mV).^[1]^

Although this was a small study with 10 rats, Delrish et al concluded that surface charge is a key factor determining vitreous diffusion and retinal penetration for chitosan-derived intraocular drug-delivery mechanisms, and they propose future use of thiolated chitosan derivatives for intravitreal chemotherapy delivery aimed at retinoblastoma treatment.^[1]^ Of relevance to the article at hand, Delrish et al recently reported improved efficacy and tumor control using thiolated chitosan nanoparticles containing topotecan compared to free topotecan in a rat model of retinoblastoma.^[18]^ These early studies indicate feasibility and potential benefit of the application of thiolated chitosan derivatives in retinoblastoma treatment. Although further study is required to investigate the safety and reproducibility prior to human trials, there are potential exciting applications of these biocompatible nanoparticles to improve treatment outcomes for retinoblastoma in the future, and this technology could also be applied to a wide variety of other retinal diseases which have traditionally been managed with intravitreal injections.

##  Financial Support and Sponsorship

This publication was supported by Grant No. P30 CA015083 from the National Cancer Institute. Its contents are solely the responsibility of the authors and do not necessarily represent the official view of the National Institutes of Health.

##  Conflicts of Interest

There are no conflicts of interest.
